# Antimicrobial Drug Use and Macrolide-Resistant *Streptococcus pyogenes*, Belgium

**DOI:** 10.3201/eid1809.120049

**Published:** 2012-09

**Authors:** Liesbet Van Heirstraeten, Samuel Coenen, Christine Lammens, Niel Hens, Herman Goossens, Surbhi Malhotra-Kumar

**Affiliations:** University of Antwerp, Antwerp, Belgium (L. Van Heirstraeten, S. Coenen, C. Lammens, N. Hens, H. Goossens, S. Malhotra-Kumar);; and University of Hasselt, Hasselt, Belgium (N. Hens)

**Keywords:** Group A streptococcus, antimicrobial resistance, fitness costs, macrolide use, macrolides, erm(A), inducible resistance, Belgium, bacteria, Streptococcus pyogenes

## Abstract

In Belgium, decreasing macrolide, lincosamide, streptogramins B, and tetracycline use during 1997–2007 correlated significantly with decreasing macrolide-resistant *Streptococcus pyogenes* during 1999–2009. Maintaining drug use below a critical threshold corresponded with low-level macrolide-resistant *S. pyogenes* and an increased number of *erm*(A)-harboring *emm*77 *S. pyogenes* with low fitness costs.

Macrolide resistance in *Streptococcus pyogenes* results primarily from modification of the drug target site by methyltransferases encoded by *erm* genes, *erm*(A) and *erm*(B) or by active efflux mediated by a *mef*-encoded efflux pump. Of these, *erm*(A) is inducibly expressed (*1*) and generally confers low-level resistance to macrolides, whereas lincosamides and streptogramins B (MLS_B_), which share overlapping binding sites, remain active against *erm*(A)-harboring *S. pyogenes* (*2*). Conversely, *erm*(B) can be constitutively or inducibly expressed and confers high-level resistance to MLS_B_ (*2*). *mef*(A) also is constitutively expressed but confers low to moderate resistance to 14- and 15-membered macrolides and susceptibility to 16-membered MLS_B_ (*2*).

That macrolide use is the main driver of macrolide resistance in streptococci has been well demonstrated at the population and individual levels (*3*,*4*). Because *erm* and *mef* are cocarried with *tet* genes on mobile elements, tetracycline use also affects macrolide resistance (*4*). In addition, acquisition of resistance often confers a cost to bacteria, the magnitude of which is the main parameter influencing the rate of development and stability of the resistance mechanisms and, conversely, the rate at which resistance would decrease under decreasing use of antimicrobial drugs (*5*). We investigated temporal changes in the molecular epidemiology of macrolide-resistant *S. pyogenes* during 1999–2009 in relation to strain fitness (i.e., ability of bacteria to survive and reproduce) and to outpatient use of MLS_B_ and tetracycline in Belgium.

## The Study

**Figure 1:**
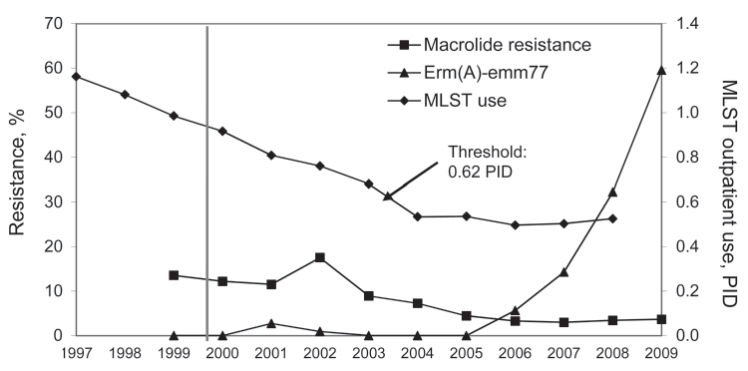
Prevalence of macrolide-resistant *Streptococcus pyogenes* and proportions of the *erm*(A)-*emm*77 geno-*emm*-type among macrolide-resistant strains during 1999–2009, and macrolides, lincosamides, streptogramins B, and tetracycline (MLST) use data expressed in packages/1,000 inhabitants/day (PID) during 1997–2007 in Belgium. Threshold indicates the critical level of macrolide, lincosamide, streptogramins B, and tetracycline use below which low-level macrolide-resistant *S. pyogenes* and selection of an inducible resistance mechanism with a lower fitness cost might be facilitated. Dotted line indicates start of the public health campaigns to reduce antimicrobial drug prescribing. The sharp increase in macrolide resistance in 2002 was linked to a local clonal outbreak of *mef(A)-emm1* harboring *S. pyogenes*.

**Figure 2:**
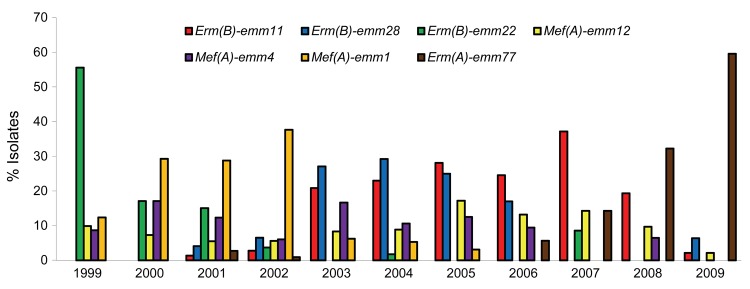
Predominant geno-*emm*-types that accounted for >5% of macrolide-resistant *Streptococcus pyogenes*, Belgium, 1999–2009.

We screened 11,819 *S. pyogenes* isolates from patients with tonsillopharyngitis or invasive disease in Belgium during 1999−2009 for macrolide resistance. We used double-disk diffusion, MIC testing, and multiplex PCR to detect *erm* and *mef* genes and investigated their clonality by *emm* typing and by pulsed-field gel electrophoresis (*6*). The prevalence of macrolide-resistant *S. pyogenes* decreased from 13.5% to 3.3% during 1999−2006 and remained low from 2006 onward (Figure 1); most isolates harbored *erm*(B) (395 [46.5%]) or *mef*(A) (383 [45.1%]). We detected e*rm*(A) in only 85 (10.0%) resistant strains; however, their proportions among macrolide-resistant strains increased from 1 (1.2%) of 81 in 1999 to 36 (76.6%) of 47 in 2009. *erm*(A)-harboring *S. pyogenes* isolates primarily belonged to *emm*77 (50/85[5.8%]). *mef*(A) was mostly associated with *emm*1, *emm*4, and *emm*12 and *erm*(B) with *emm*11, *emm*22, and *emm*28 (Figure 2). During 1999−2009, proportions of *mef*(A)- and *erm*(B)-associated *emm* types decreased gradually, whereas those of *erm*(A)-harboring *emm*77 (*erm*(A)-*emm*77) )increased steadily from 2006 onward (Figure 2). *erm*(A)-*emm*77 became predominant in 2008−2009, representing 10−28 (32.2%−59.6%) of total macrolide-resistant *S. pyogenes* isolates during those 2 years (Figure 1, Figure 2). Most (97.8%) *erm(A)-emm*77 belonged to the same pulsed-field gel electrophoresis cluster and harbored *tet*(O), indicating gene linkage.

Next, we used data on outpatient use of MLS_B_ and tetracycline collected by the Belgian National Institute for Health and Disability Insurance during 1997−2008 and aggregated at the active substance level (World Health Organization Collaborating Center for Drug Statistics Methodology, www.whocc.no/atc/structure_and_principles/) to model the data obtained for macrolide-resistant *S. pyogenes*. MLS_B_ and tetracycline use was expressed in packages/1,000 inhabitants/day, a better proxy for prescriptions than defined daily doses in Belgium, where the number of defined daily doses per package or prescription had increased during the previous decade (*7*). MLS_B_ and tetracycline use decreased from 1997 to 2004 (1.16–0.53 packages/1,000 inhabitants/day) and remained stable at this level (0.50−0.53 packages/1,000 inhabitants/day) from 2004 onward (Figure 1). Total outpatient use of antimicrobial drugs also decreased (3.75–2.4 packages/1,000 inhabitants/day) during 1997–2007, as did use of penicillins, whereas proportional use of amoxicillin–clavulanate acid increased transiently soon after public campaigns began in Belgium (*8*). Yearly proportions of macrolide-resistant strains among total isolates correlated with MLS_B_ and tetracycline use in generalized linear models with a negative binomial distribution and a log-link (GLM, PROC GENMOD, SAS Institute, Cary, NC, USA). Using an interval of 2 years, we observed a highly significant positive correlation between decreasing use of MLS_B_ and tetracycline during 1997−2007 and decreasing levels of macrolide-resistant *S. pyogenes* during 1999−2009 (p<0.0001). The consistent decrease in MLS_B_ and tetracycline use since 1997 was further accentuated by the start of public health campaigns in December 2000 that also were directed toward prescribers and successfully reduced antimicrobial drug prescribing in Belgium (Figure 1) (*8*). A similar trend was observed in Finland, where a nationwide increase in erythromycin use and resistant *S. pyogenes* led to issuance of national recommendations to reduce outpatient use of MLS_B_; erythromycin-resistant *S. pyogenes* declined after 2 years of reduced MLS_B_ use (*9*). Nonetheless, for *S. pyogenes*, these correlations are not always clear, primarily because of frequent clonal fluctuations for this organism. For instance, despite a 21% decrease in macrolide use in Slovenia, resistance doubled among noninvasive *S. pyogenes* isolates (*10*).

Notwithstanding clonal changes, the fitness costs (i.e., an organism’s decreased ability to survive and reproduce because of a genetic change, expressed as a decreased bacterial growth rate) associated with particular resistance mechanisms is another major factor governing the relation between use and resistance. Mathematical models have shown threshold levels of antimicrobial drug use below which the frequency of resistance would not increase if resistance imposes a fitness cost for the bacteria (*11*). We further hypothesized that the frequency of certain macrolide-resistant geno-*emm*-types might differ if antimicrobial drug use remains below a certain threshold. In concordance with the models, we found a negative correlation between use of MLS_B_ and tetracycline and proportions of *erm*(A)-*emm*77 among macrolide-resistant *S. pyogenes* (p = 0.0002), and we identified 0.62 packages/1,000 inhabitants/day as the critical threshold volume of MLS_B_ and tetracycline use below which proportions of *erm*(A)-*emm*77 among macrolide-resistant *S. pyogenes* would increase significantly (p<0.0001). Next, we compared the fitness of *erm*(A)-*emm*77 with that of 6 other major macrolide-resistant geno-*emm*-types in Belgium during 1999–2009 (Figure 2). After growth-competition experiments (*12*), initial and final proportions of competing strains were determined by multiplex PCR to detect *erm*(B), *erm*(A), or *mef*(A) in 50 randomly selected colonies per plated mixture. Number of generations and relative fitness of competed pairwise strains were calculated as described (*13*). The inducible *erm*(A) in an *emm*77 background was more fit (67%) than most of the geno-*emm*-types that predominated during the previous years of higher MLS_B_ and tetracycline use (Table). Only the *mef*(A)-*emm*1 and *erm*(B)-*emm*28 geno-*emm*-types were equally as fit as *erm*(A)-*emm*77. Foucault et al. (*14*) showed that in the noninduced state, the inducible *vanB* gene had no effect on fitness of enterococci and might explain the low fitness cost of *erm*(A) carriage in *emm*77 strains. A predominance of *erm*(A)-harboring strains during 1993–2002, with 30% in an *emm77* background, was also reported in Norway, a country with a low prevalence of resistance (2.7%) and antimicrobial drug use (*15*). Of note here is the combination of *erm*(A) and *emm*77 as geno-*emm*-type because the fitness benefit (i.e., lack of fitness cost) was not as remarkable for other *erm*(A)-harboring *emm* types (data not shown). The mechanisms underlying the higher fitness benefit conferred by an *emm*77 versus another *emm* background for the *erm*(A) genetic element remain to be investigated and might be related to differences in basal gene expression or compensatory changes in the *emm*77 genome or might result from differences in the genetic element harboring *erm*(A) in *emm*77.

**Table:** Characteristics of macrolide-resistant *Streptococcus pyogenes* used in competition experiments and relative fitness* of the *erm*(A)-*emm*77 geno-*emm*-type against competitor strains, Belgium, 1999–2009

Geno-*emm*-type	Macrolide MIC, mg/L			Relative fitness (SD)†		p value, *t* test
	Erythromycin	Clindamycin		*erm(A)-emm77*	Competitor	
*erm*(A)-*emm77*	2	0.125		ND	ND	ND
*erm*(B)-*emm28*	>512	>512		1.03 (0.09)	0.98 (0.09)	0.662
*erm*(B)-*emm22*	>512	>512		1.27 (0.15)	0.79 (0.09)	0.080
*erm*(B)-*emm11*	>512	256		2.12 (0.28)	0.48 (0.06)	0.013
*mef*(A)-*emm12*	8	0.125		1.29 (0.18)	0.78 (0.11)	0.105
*mef*(A)-*emm4*	8	<0.03		1.55 (0.15)	0.65 (0.06)	0.047
*mef*(A)-*emm1*	16	0.5		1.01 (0.05)	1.00 (0.06)	0.934

*The ability of the bacteria to survive and reproduce. ND, no data. †Average of duplicate experiments.

## Conclusions

Using macrolide-resistant *S. pyogenes* as a marker for use of MLS_B_ and tetracycline, we showed a decrease in use of these antimicrobial drugs, accentuated by successful public health campaigns, reflected a steady decline of macrolide-resistant *S. pyogenes* in Belgium. Furthermore, successfully maintaining use below a critical threshold resulted in maintenance of low-level macrolide-resistant *S. pyogenes* and emergence of the inducibly expressed and low-level resistant *erm*(A)-*emm*77 geno-*emm*-type. Maintaining antimicrobial drug use below a critical threshold might facilitate stabilization of low-level antimicrobial drug resistance and of milder resistance mechanisms with lower fitness costs.
